# Comparative changes in problematic smartphone use tendencies across four active exercise modalities among college students: an exploratory multicategorical relative indirect-effect analysis

**DOI:** 10.3389/fpsyg.2026.1896277

**Published:** 2026-09-03

**Authors:** Bin Dai, Canzhong Ji, Tingting Zhong

**Affiliations:** 1 School of Sports Science and Physical Education, Nanjing Normal University, Nanjing, Jiangsu, China; 2 School of Physical Education and Health Preservation, Communication University of Kunming, Kunming, Yunnan, China

**Keywords:** emotion regulation, exercise intervention, mediation effect, randomized controlled trial, smartphone addiction

## Abstract

**Objective:**

To compare pre–post changes in problematic smartphone use tendencies and emotion regulation across four exercise modalities and examine whether cognitive reappraisal and expressive suppression changes accounted for between-modality differences in SAS-SV reduction.

**Methods:**

Ninety college students were assessed; five were excluded before randomization for exercise contraindications, and 85 were randomized to aerobic training, strength training, VR-based gamified exercise, or combined training. Seventy-seven participants completing both assessments and at least 80% of sessions entered a modified per-protocol complete-case analysis; no intention-to-treat analysis was conducted. All groups completed a 12-week intervention. Outcomes were assessed pre and post-intervention. Group differences were examined using mixed-design ANOVA, followed by exploratory multicategorical parallel mediation with aerobic training as the reference.

**Results:**

SAS-SV scores and expressive suppression decreased, whereas cognitive reappraisal increased, in all groups. Significant time × group interactions occurred for SAS-SV, *F*(3, 73) = 45.205, *p* < 0.001, partial η^2^ = 0.650; cognitive reappraisal, *F*(3, 73) = 21.992, *p* < 0.001, partial η^2^ = 0.475; and expressive suppression, *F*(3, 73) = 18.860, *p* < 0.001, partial η^2^ = 0.437. The combined group showed the largest changes. Relative to aerobic training, total indirect effects were 0.722 for strength training, 1.253 for VR-based exercise, and 1.916 for combined training; mediator-specific indirect effects were nonzero for all contrasts.

**Conclusion:**

The four modalities were associated with different magnitudes of pre–post change, with combined training showing the largest changes. Without diagnostic selection or a non-exercise control, the findings concern problematic smartphone use tendencies in a college-student sample and do not establish exercise-specific effects or causal mediation.

## 1 Introduction

With the development of the mobile Internet, smartphones have become embedded in college students’ academic, social, and leisure activities and increasingly serve emotion regulation and stress-relief functions. Although the term “smartphone addiction” is widely used in the literature and appears in the official name of the SAS-SV, it does not represent a uniformly established clinical diagnosis, and its conceptual boundaries remain debated. In the present study, the term “problematic smartphone use tendencies” refers to continuous SAS-SV scores reflecting excessive, difficult-to-control smartphone use and related functional difficulties. It does not indicate that participants met diagnostic criteria for a behavioral addiction. College students are at a critical developmental stage characterized by identity formation, expanding social relationships, and worldview development, making them particularly susceptible to academic pressure, employment-related anxiety, loneliness, and the digital media environment. When smartphone use shifts from an instrumental need to addictive dependence, individuals may use social media, short videos, online games, and related applications to escape real-life stress, thereby increasing the risk of problematic smartphone use and leading to attentional distraction, poor sleep quality, and impaired academic performance (Busch and McCarthy, 2021; Cheng et al., 2024; Ratan et al., 2021).

Emotion regulation is central to understanding problematic smartphone use tendencies and refers to psychological processes through which individuals influence the occurrence, duration, and expression of emotions, including the identification, monitoring, and modification of emotional generation, experience, and expression; its core function is to help individuals cope with stress, alleviate negative emotions, and maintain psychological adjustment (Gross, 1998). Among college students, Cheng et al. (2024) found that daily negative emotional experiences were positively associated with smartphone addiction, with stress playing an important mediating role. From a network analysis perspective, Cho and Kim (2025) further identified emotional dysregulation and impulsivity as important psychological nodes in smartphone overdependence. Among different emotion regulation strategies, cognitive reappraisal is generally regarded as a more adaptive strategy that helps individuals reinterpret stressful events, whereas expressive suppression involves controlling outward emotional responses and may reinforce emotional avoidance and psychological burden (Gross and John, 2003; Rozgonjuk and Elhai, 2021).

Existing interventions targeting problematic smartphone use and related forms of problematic digital-media use include cognitive behavioral therapy, digital use management, psychoeducation, and mindfulness training. Although these approaches can improve self-monitoring, use control, and emotional coping, their implementation in universities is constrained by limited resources, lengthy intervention periods, and demanding standardization requirements. In contrast, physical exercise is low-cost, organized, and suitable for campus settings. Studies have reported negative associations between physical activity and problematic smartphone use indicators among college students while meta-analyses of adolescent mobile phone addiction indicate that exercise interventions can reduce addiction levels, with effects varying by exercise type, intensity, and frequency (Chen et al., 2025; Li et al., 2023). Accordingly, changes in emotion regulation may be statistically associated with changes in SAS-SV scores and may help characterize differences across exercise modalities.

The selection of aerobic training, strength training, VR-based gamified exercise, and combined training was grounded in the theoretical logic of “behavioral substitution–self-regulation–reward feedback–mechanistic integration.” Aerobic training is low-cost and easy to implement and may serve as a basic intervention by increasing physical activity, reducing smartphone use, and improving emotional states; strength training features clear goals, progressive overload, and task completion, which may enhance bodily control, self-efficacy, and emotion regulation; VR-based gamified exercise, through immersive environments, interactive tasks, and immediate feedback, can maintain exercise load while enhancing enjoyment, motivation, and positive emotional experiences. Combined training integrates the structured advantages of strength training with the immersive feedback features of VR-based gamified exercise and may produce complementary effects in behavioral substitution, self-regulation, and reward restructuring. Because these modalities differ in task structure, feedback characteristics, and experiential components, the patterns of change in emotion-regulation outcomes were expected to differ across modalities. A systematic review of exergames has shown that they can increase positive emotional experiences and may serve as an activity format for promoting well-being and emotion regulation (Marques et al., 2023). Meanwhile, VR and gamification-based interventions have also shown potential for managing mental health problems such as anxiety and depression (Jingili et al., 2023). A recent randomized controlled trial of immersive VR-based exercise among college students indicated that VR exercise may improve certain psychological and physiological health indicators, providing empirical support for its application in mental health promotion in university settings (Liu et al., 2025).

It should be noted that existing findings on the relationship between physical exercise and problematic smartphone use are not entirely consistent. Although most studies support a negative association between physical activity levels and problematic smartphone use, some studies have reported weak associations or unstable findings across different populations. Relevant reviews have further indicated that the current evidence is still dominated by cross-sectional studies, and substantial heterogeneity exists across studies in terms of exercise type, intervention duration, exercise intensity, measurement tools, and sample characteristics, which may contribute to inconsistent or mixed intervention effects (Chen et al., 2025; Li et al., 2023; Pirwani and Szabo, 2024, 2025). In addition, changes in problematic smartphone use associated with exercise participation may not depend solely on exercise participation itself but may be jointly shaped by exercise modality, contextual experience, feedback mechanisms, individual motivation, and emotion regulation capacity.

Based on the above evidence, this study adopted an active-comparator randomized design and incorporated traditional aerobic training, strength training, VR-based gamified exercise, and combined training into a unified analytical framework to compare pre–post changes in problematic smartphone use tendencies across the four active intervention conditions. An exploratory multicategorical parallel mediation analysis was further conducted to examine whether changes in cognitive reappraisal and expressive suppression statistically accounted for relative between-modality differences in reductions in SAS-SV scores. Accordingly, the following hypotheses and exploratory research question were proposed: first, SAS-SV scores would decrease from pre to post-intervention within all four active conditions; second, strength training, VR-based gamified exercise, and combined training would show larger reductions in SAS-SV scores than traditional aerobic training, with the combined condition expected to show the largest reduction; third, as an exploratory research question, we examined whether changes in cognitive reappraisal and expressive suppression statistically accounted for the relative intervention contrasts in SAS-SV score reduction.

## 2 Methods

### 2.1 Participants

No diagnostic interview, clinical diagnostic criteria, or SAS-SV screening cutoff was used for recruitment. Therefore, SAS-SV scores were treated as continuous indicators of problematic smartphone use tendencies, and the participants should be interpreted as a general college-student sample rather than as individuals with clinically diagnosed smartphone addiction. Recruitment eligibility criteria were specified before randomization. The inclusion criteria were as follows: (1) being a full-time college student at the participating university; (2) being able to safely participate in moderate-intensity physical exercise, with no apparent contraindications to exercise based on course-based health records and pre-participation screening; (3) using a smartphone in daily life and being able to complete the Smartphone Addiction Scale–Short Version and Emotion Regulation Questionnaire; and (4) voluntarily agreeing to participate and providing written informed consent. The exclusion criteria for recruitment were as follows: (1) having cardiovascular, respiratory, neurological, vestibular, or musculoskeletal conditions that could make participation in the exercise intervention unsafe; (2) having recently participated in another structured psychological intervention, exercise intervention, or smartphone-use management program that could influence the study outcomes; and (3) experiencing severe visual discomfort, dizziness, or intolerance to VR equipment. Habitual exercise level, formal mental health status, and previous VR experience were not used as eligibility criteria because these characteristics were not systematically assessed at baseline. The primary analytic population was defined using a modified per-protocol complete-case criterion. Randomized participants were included in the primary analysis if they completed both the baseline and post-intervention assessments and attended at least 80% of the scheduled intervention sessions. The attendance threshold was selected to ensure adequate exposure to the assigned 12-week exercise protocol. Participants who did not complete the post-intervention assessment or did not meet the prespecified attendance threshold were excluded from the primary analysis. Therefore, the primary analysis did not follow the intention-to-treat principle and should be interpreted as an analysis of adherent completers rather than of all randomized participants. Illness-related absence and transfer from the university were documented as reasons contributing to incomplete follow-up or insufficient attendance.

**Figure 1:**
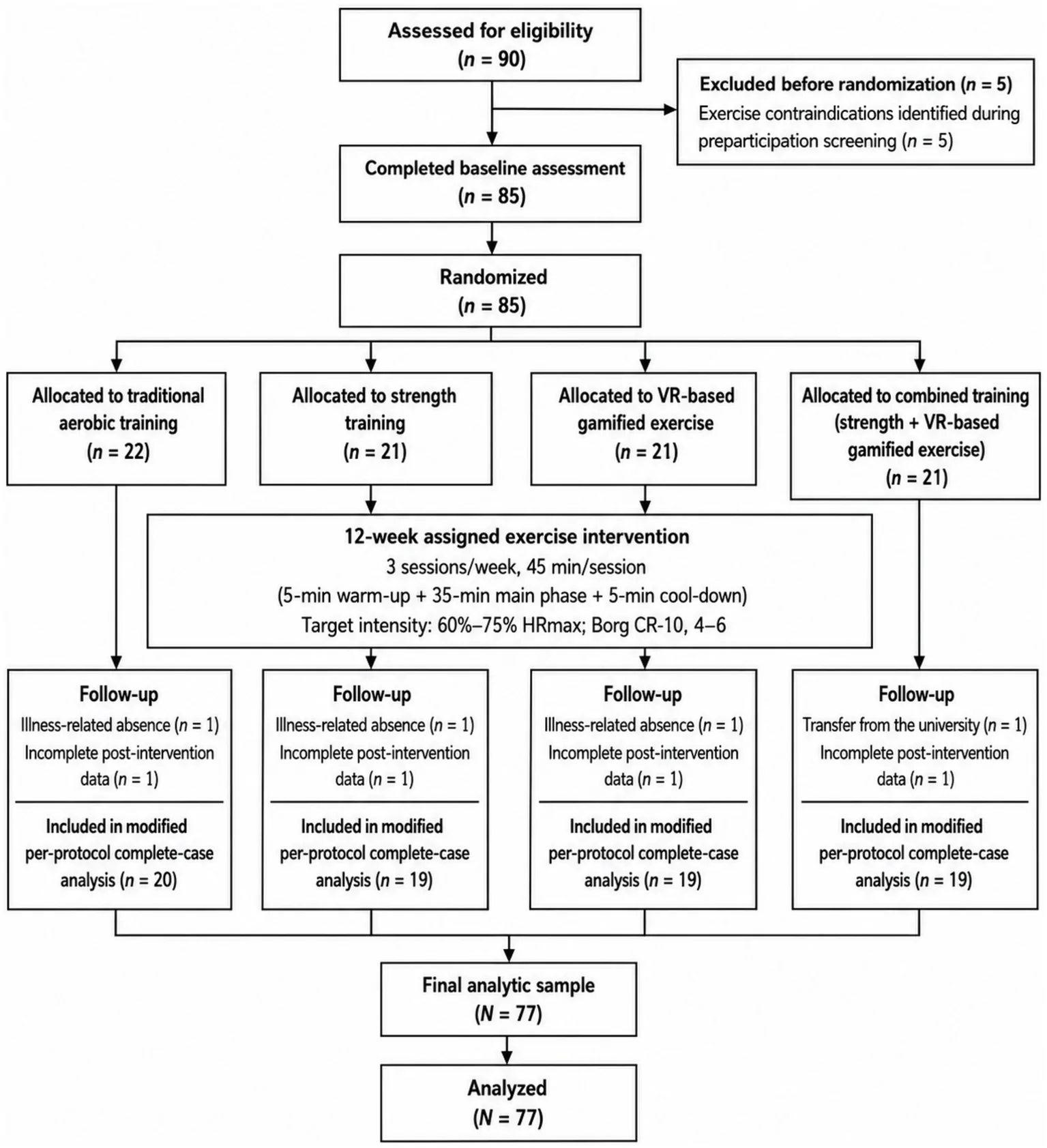
CONSORT flow diagram of participants.

Before the formal intervention, the researchers explained the study purpose, implementation procedures, potential risks, and right to withdraw to all participants, and written informed consent was obtained after they had been fully informed. This study was approved by the Ethics Committee of Nanjing Normal University (Approval No. NJNU20260329), and all procedures were conducted in accordance with the Declaration of Helsinki (World Medical Association, 2025). An *a priori* sample size calculation for repeated-measures analysis of variance was performed using G*Power 3.1 (Faul et al., 2009), with the effect size set at *f* = 0.25, the significance level at *α* = 0.05, and statistical power at 1—*β* = 0.80; under a design involving four groups and two measurement time points, the minimum required sample size was calculated to be 48 participants, and after accounting for an anticipated dropout rate of approximately 10%, at least 53 participants were required for recruitment. To account for potential attrition, 85 participants were randomized, exceeding the minimum required sample size. The primary reason for each post-randomization exclusion was classified as incomplete post-intervention data, illness-related absence, or transfer from the university, as shown in Figure 1.

### 2.2 Study design

A longitudinal active-comparator randomized trial was conducted. Participants were randomly assigned to one of four groups: Group I, traditional aerobic training; Group II, strength training; Group III, VR-based gamified exercise; or Group IV, combined training consisting of strength training plus VR-based gamified exercise. Randomization was performed using a computer-generated random sequence created in Microsoft Excel by an independent statistician. To ensure allocation concealment, group assignments were placed in sequentially numbered, opaque, sealed envelopes. After participants completed baseline assessments and provided written informed consent, the study coordinator opened the next envelope in sequence to confirm group allocation. Owing to the nature of the exercise interventions, training staff were inevitably unblinded after allocation; however, outcome assessors and data analysts remained blinded to group assignment throughout the trial. Of the 90 students initially assessed, five were excluded before randomization because health-related concerns identified during pre-participation screening precluded their safe participation in the exercise interventions. The remaining 85 participants completed the baseline assessment and were randomly allocated to the traditional aerobic training group (*n* = 22), strength training group (*n* = 21), VR-based gamified exercise group (*n* = 21), or combined training group (*n* = 21). During follow-up, eight participants were excluded from the modified per-protocol complete-case analysis because of incomplete post-intervention data (*n* = 4), illness-related absence (*n* = 3), or transfer from the university (*n* = 1). Exclusions were balanced across groups, leaving 77 participants for analysis: 20 in the traditional aerobic group and 19 in each of the other groups. All four groups completed the corresponding 12-week exercise intervention during the same academic year and under comparable teaching and living conditions.

This study used a four-arm active-comparator randomized design in which all participants received an exercise intervention. Randomization supported comparisons of relative pre–post changes among the four exercise modalities; however, because no non-exercise, wait-list, or attention-control condition was included, the design could not isolate the effect of exercise per se from repeated-measurement effects, participant expectations, researcher attention, social interaction, or natural changes over time. Problematic smartphone use tendencies, cognitive reappraisal, and expressive suppression were assessed at baseline and immediately after the intervention. The procedures for participant screening, allocation, follow-up, and analysis are shown in Figure 1. Importantly, no injuries or adverse events occurred during the 12-week training.

### 2.3 Assessments

#### 2.3.1 Anthropometry

At baseline, participants’ height and body weight were measured in a standardized manner by trained research staff following the standard procedures used in physical fitness testing at Chinese universities. Height was measured using a standing stadiometer routinely used for campus physical fitness testing, with readings recorded to the nearest 0.1 cm; body weight was measured using a standard electronic scale, with readings recorded to the nearest 0.1 kg (Jiang et al., 2024). To minimize measurement error, all assessments were scheduled, where possible, during the same morning time period, and participants were instructed to avoid vigorous exercise and excessive fluid intake before testing; during measurement, they were required to remove their shoes, wear light clothing, and stand naturally. For height measurement, participants stood with their feet together, trunk upright, and eyes facing forward, and the value was recorded after the body position was stabilized; for body weight measurement, participants stood in the center of the electronic scale, and the result was recorded after the reading became stable. After the measurements were completed, body mass index (BMI) was calculated from height and body weight using the following formula: BMI = body weight (kg)/height^2^ (m^2^) (Li et al., 2025). This measurement approach required only simple equipment commonly used in university physical fitness testing, followed standardized and easily implemented procedures, was suitable for campus-based research settings, and helped ensure the authenticity and reproducibility of the data.

#### 2.3.2 Problematic smartphone use tendencies

Problematic smartphone use tendencies were assessed using the Smartphone Addiction Scale–Short Version (SAS-SV), developed by Kwon et al. (2013). Although the term “addiction” is retained in the instrument’s official title, the present study used the SAS-SV total score as a continuous indicator of problematic smartphone use tendencies rather than as a diagnostic classification. This scale was derived from the original Smartphone Addiction Scale and serves as a brief assessment tool designed to evaluate tendencies related to excessive smartphone use, withdrawal symptoms, tolerance, and loss of control, making it suitable for use among adolescents and college students. The SAS-SV consists of 10 items rated on a 6-point Likert scale, ranging from 1 = strongly disagree to 6 = strongly agree. All items are scored in the same direction, with total scores ranging from 10 to 60; higher scores indicate stronger problematic smartphone use tendencies. Example items include “missing planned work due to smartphone use” and “constantly thinking about the smartphone even when not using it.” Previous research has demonstrated that the scale has good reliability and validity, with high internal consistency (Cronbach’s α = 0.91), and can be used to assess individual differences in problematic smartphone use tendencies (Kwon et al., 2013). In the present study, the internal consistency of the scale was Cronbach’s α = 0.896.

#### 2.3.3 Emotion regulation scale

Emotion regulation was measured using the Emotion Regulation Questionnaire (ERQ), developed by Gross and John (2003). Developed based on the process model of emotion regulation, the ERQ is primarily used to assess individuals’ tendencies to use different emotion regulation strategies in daily life and includes two dimensions: cognitive reappraisal and expressive suppression. Cognitive reappraisal refers to regulating emotional experience by changing one’s cognitive interpretation of a situation, whereas expressive suppression refers to controlling or inhibiting outward emotional expression after an emotion has been generated (Gross and John, 2003). The scale consists of 10 items rated on a 7-point Likert scale, ranging from 1 = strongly disagree to 7 = strongly agree. The cognitive reappraisal subscale includes 6 items, and the expressive suppression subscale includes 4 items. Item scores were summed separately for each subscale, with higher scores indicating a greater tendency to use the corresponding emotion regulation strategy. Example items include “When I want to feel more positive emotions, I change the way I am thinking about the situation” and “I control my emotions by not expressing them.” Previous research has shown that the ERQ has good reliability and validity and is suitable for assessing stable individual differences in the use of cognitive reappraisal and expressive suppression strategies (Gross and John, 2003). In the present study, the internal consistency of the scale was Cronbach’s *α* = 0.915.

### 2.4 Intervention programs

The intervention period lasted 12 weeks, with training frequency and duration standardized across the four groups: three sessions per week, 45 min per session, and a total weekly training duration of 135 min; each session consisted of a 5-min warm-up, 35 min of main training, and 5 min of cool-down and stretching.

#### 2.4.1 Traditional aerobic training group

The traditional aerobic training group used continuous paced jogging as the fixed intervention modality. Each 45-min session consisted of a 5-min dynamic warm-up, 35 min of paced jogging, and a 5-min cool-down. Running speed was adjusted according to real-time heart-rate responses and perceived exertion, with prescribed targets of 60–75% of HRmax and Borg CR-10 ratings of 4–6 (Borg, 1982; Garber et al., 2011). Speed was increased when heart rate fell below the target range and reduced, or briefly replaced with brisk walking, when heart rate exceeded the target range. Before and during each session, participants were monitored for discomfort, gait instability, excessive fatigue, and other adverse responses. Training intensity was reduced or the session was discontinued if marked shortness of breath, chest tightness, dizziness, or lower-limb pain occurred. Although heart-rate responses, Borg ratings, training duration, and discomfort symptoms were monitored during intervention delivery, these session-level records were not sufficiently complete and standardized for group-level quantitative analysis.

#### 2.4.2 Strength training group

The strength training group completed a standardized circuit resistance-training program. Each 45-min session consisted of a 5-min warm-up, 35 min of circuit training, and a 5-min cool-down and stretching period. The main training component included eight fixed exercises—squats, lunges, push-ups, resistance-band rows, abdominal crunches, planks, glute bridges, and dumbbell shoulder presses—performed for 40 s each with 20-s transitions. Participants completed four circuits with 1–2 min of rest between circuits. Training followed progressive-overload principles, with load adjusted according to heart-rate responses, Borg CR-10 ratings, movement quality, and individual capacity (Kraemer and Ratamess, 2004). Participants maintained Borg CR-10 ratings of 4–6 and approximately 2–3 repetitions in reserve to avoid training to failure (Zourdos et al., 2016). Resistance, repetitions, or rest intervals were adjusted if movement technique deteriorated or participants experienced joint pain, loss of movement control, or excessive fatigue. Although training load, completed rounds and repetitions, heart-rate responses, Borg ratings, muscle soreness, and adverse responses were monitored during intervention delivery, these session-level records were not sufficiently complete and standardized for group-level quantitative analysis.

#### 2.4.3 VR-based gamified exercise group

The VR-based gamified exercise group used a PICO Neo3 standalone headset and fixed rhythm and boxing-based games, including Synth Riders and FitXR. Each 45-min session consisted of a 5-min warm-up, 35 min of VR-based exercise, and a 5-min cool-down. The main exercise phase included 15–17 min of rhythm-based exercise, a 1–2-min rest interval, and 15–17 min of boxing-based exercise. A 1-week familiarization period was provided before the formal intervention, during which game difficulty was gradually increased from low to moderate; thereafter, the game types, training sequence, and session duration were standardized. Game difficulty, song speed, movement amplitude, or rest intervals were adjusted according to real-time heart-rate responses and Borg CR-10 ratings to target 60–75% of HRmax and Borg scores of 4–6. Headset fit, controller security, and activity-area safety were checked before each session, and participants were monitored for dizziness, nausea, visual fatigue, balance instability, and spatial-orientation difficulties. Training was paused if marked VR-related discomfort or postural instability occurred. Although effective exercise time, heart-rate responses, Borg ratings, game settings, perceived enjoyment, and discomfort symptoms were monitored during intervention delivery, these session-level records were not sufficiently complete and standardized for group-level quantitative analysis.

#### 2.4.4 Combined training group: strength training plus VR-based gamified exercise

The combined training group integrated circuit resistance training with VR-based gamified exercise. Each 45-min session consisted of a 5-min warm-up, approximately 16–18 min of strength training, 16–18 min of VR-based exercise, and a 5-min cool-down. The strength component comprised two circuits using the same eight-exercise pool as the strength training group, whereas the VR component used the same headset, games, and difficulty-adjustment procedures as the VR-based gamified exercise group. Component durations were shortened to maintain the same prescribed session duration as the other groups. The target intensity was 60–75% of HRmax and Borg CR-10 ratings of 4–6, with movement tempo, resistance, VR-game difficulty, transition time, or rest adjusted according to heart-rate responses, perceived exertion, movement quality, and participant symptoms. Movement quality, muscular fatigue, and joint stability were monitored during strength training, whereas VR-related discomfort, balance, and visual fatigue were monitored during the VR component. Although component completion, heart-rate responses, target-zone time, Borg ratings, and discomfort symptoms were monitored during intervention delivery, the session-level records were insufficient to quantitatively verify achieved exercise-dose or intensity comparability across groups.

#### 2.4.5 Intervention delivery, pre-specified monitoring procedures, and available fidelity data

Before the intervention, a standardized operations manual was developed in accordance with the Consensus on Exercise Reporting Template recommendations (Slade et al., 2016). The manual specified the training content, session structure, target intensity, progression principles, safety precautions, and monitoring procedures for each condition. All sessions were supervised on site by trained researchers or physical education professionals who had received standardized training in protocol delivery, movement technique, VR-device operation, safety monitoring, and session documentation. A predefined checklist was used to support consistent completion of the warm-up, main exercise component, cool-down, intensity monitoring, and safety procedures. Heart-rate responses, Borg CR-10 ratings, movement quality, exercise completion, and discomfort symptoms were used operationally to guide real-time adjustments in exercise pace, resistance, rest intervals, or VR-game difficulty.

Attendance and adverse-event records were the only fidelity indicators retained in a sufficiently complete and standardized format for quantitative analysis. Although session-level heart-rate responses, Borg CR-10 ratings, time within the target heart-rate zone, and training-completion indicators were monitored during intervention delivery, these records were insufficient for group-level summary or between-group comparison. Therefore, the intensity ranges and exercise doses reported in Table 1 should be interpreted as prespecified protocol targets rather than quantitatively verified achieved values, and equivalence of actual exercise intensity or dose across the four groups could not be established.

**Table 1:** Prescribed training protocols and planned monitoring procedures for the four intervention groups.

Group	Prescribed training content	Prescribed dose, target intensity, and planned monitoring
Group I traditional aerobic training	Type: continuous paced jogging. Session structure: 5-min dynamic warm-up, 35-min paced jogging, and 5-min cool-down and stretching. Setting: Campus track/playground.	Frequency and duration: 3 sessions/week for 12 weeks; 45 min/session; prescribed dose, 135 min/week. Target intensity: 60–75% HRmax; target Borg CR-10, 4–6. Planned real-time adjustment: Running speed adjusted using heart-rate responses and perceived exertion; brief brisk walking permitted for recovery. Safety monitoring: Discomfort symptoms, gait stability, and signs of excessive fatigue monitored during delivery.
Group II strength training	Type: standardized circuit resistance training. Exercises: Squats, lunges, push-ups, resistance-band rows, abdominal crunches, planks, glute bridges, and dumbbell shoulder presses. Format: 40 s exercise/20 s transition; 4 circuits; 1–2 min rest between circuits. Setting: Campus gym/training area.	Frequency and duration: 3 sessions/week for 12 weeks; 45 min/session; prescribed dose, 135 min/week. Target intensity: Target Borg CR-10, 4–6; approximately 2–3 repetitions in reserve. Planned real-time adjustment: Heart-rate responses, perceived exertion, movement quality, and symptoms used to adjust resistance, repetitions, or rest. Safety monitoring: Technique deterioration, joint pain, and excessive fatigue monitored during delivery.
Group III VR-based gamified exercise	Type: immersive rhythm and boxing-based VR exercise. Equipment/games: PICO Neo3; Synth Riders and FitXR. Main phase: 15–17 min rhythm exercise, 1–2 min rest, and 15–17 min boxing exercise. Familiarization: 1 week before the formal intervention. Setting: Supervised indoor VR area.	Frequency and duration: 3 sessions/week for 12 weeks; 45 min/session; prescribed dose, 135 min/week. Target intensity: 60–75% HRmax; target Borg CR-10, 4–6. Planned real-time adjustment: Game difficulty, song speed, movement amplitude, or rest adjusted using heart-rate responses and perceived exertion. Safety monitoring: Cybersickness, visual fatigue, balance instability, and spatial-orientation difficulties monitored during delivery.
Group IV combined training (strength + VR)	Type: circuit resistance training plus VR-based gamified exercise. Strength component: 2 circuits using the same 8-exercise pool as Group II (approximately 16–18 min). VR component: Same equipment and games as Group III (approximately 16–18 min). Session design: Component durations were shortened to preserve the common 35-min main phase. Setting: Campus gym and supervised VR area.	Frequency and duration: 3 sessions/week for 12 weeks; 45 min/session; prescribed dose, 135 min/week. Target intensity: 60–75% HRmax; target Borg CR-10, 4–6. Planned real-time adjustment: Movement tempo, resistance, VR-game difficulty, transition time, or rest adjusted using heart-rate responses, perceived exertion, movement quality, and symptoms. Safety monitoring: Accumulated fatigue, joint stability, cybersickness, balance, and visual fatigue monitored during delivery.

The intensity ranges, exercise durations, and monitoring indicators represent prespecified intervention targets and delivery procedures. Session-level heart-rate responses, Borg CR-10 ratings, time within the target heart-rate zone, and training-completion records were not sufficiently complete and standardized for group-level analysis. Therefore, achieved exercise intensity and dose comparability across groups could not be quantitatively verified. Only attendance and adverse-event data were available for quantitative fidelity reporting. HRmax, maximum heart rate; Borg CR-10, Borg category-ratio 10 scale; VR, virtual reality.

### 2.5 Statistical analysis

Statistical analyses were conducted using SPSS 27.0, and mediation effects were examined using the PROCESS macro for SPSS (Hayes and Rockwood, 2017). Continuous variables are presented as means ± standard deviations, whereas categorical variables are presented as frequencies and percentages; the significance level was set at a two-tailed *p* < 0.05. Before the formal analyses, data completeness, univariate outliers, and statistical assumptions were examined. The primary analyses were conducted using a modified per-protocol complete-case approach rather than an intention-to-treat approach. The primary analysis was restricted to participants who completed both assessments and attended at least 80% of the scheduled intervention sessions. An intention-to-treat analysis was not conducted because post-intervention outcome data were unavailable for some randomized participants, the original analysis plan required adequate exposure to the assigned intervention, and no likelihood-based or multiple-imputation procedure had been pre-specified for handling missing post-intervention outcomes. Rather than introducing *post hoc* imputed values based on assumptions that could not be adequately verified in this relatively small sample, the analyses were restricted to participants meeting the pre-specified follow-up and adherence criteria. No values were missing for SAS-SV scores, cognitive reappraisal, or expressive suppression among the 77 participants included in the final analytic dataset. Consequently, the reported estimates apply primarily to adherent completers rather than to all randomized participants. The potential influence of this analytic decision on internal validity and generalizability is addressed in the Limitations section.

Univariate outliers were screened using boxplots and standardized Z scores. Observations outside 1.5 times the interquartile range were considered mild outliers, whereas |Z| > 3.29 was used as the criterion for an extreme univariate outlier. All flagged values were checked against the theoretical ranges of the corresponding scales and the original data records. Normality was assessed using the Shapiro–Wilk test within each intervention group for the pre-test, post-test, and change scores (Shapiro and Wilk, 1965). Homogeneity of variance across the four groups was examined using the median-centered Levene test (Brown and Forsythe, 1974).

Separate 4 × 2 mixed-design analyses of variance were conducted for SAS-SV scores, cognitive reappraisal, and expressive suppression. The main effects of time and group and the time × group interaction were examined, with the interaction treated as the primary effect of interest. Because the within-subject factor time consisted of only two levels, the assumption of sphericity was satisfied by definition (*ε* = 1.000); therefore, Mauchly’s test and the Greenhouse–Geisser and Huynh–Feldt corrections were not applicable (Keselman et al., 2001).

When the homogeneity-of-variance assumption was violated for cross-sectional between-group comparisons, Welch’s analysis of variance was used (Delacre et al., 2019). When change scores deviated significantly from normality, Wilcoxon signed-rank tests were conducted as sensitivity analyses for within-group pre–post comparisons, and Kruskal–Wallis H tests were conducted as sensitivity analyses for overall between-group comparisons of change scores. Significant time × group interactions were followed by paired-samples t tests and Bonferroni-adjusted comparisons of change scores. The non-parametric analyses were used to assess the robustness of the overall conclusions rather than to replace the primary mixed-design analyses.

Change scores were coded so that higher values consistently represented greater improvement. Reduction in SAS-SV scores was calculated as the pre-intervention score minus the post-intervention score (ΔSAS = pre − post); improvement in cognitive reappraisal was calculated as the post-intervention score minus the pre-intervention score (ΔCR = post − pre); and reduction in expressive suppression was calculated as the pre-intervention score minus the post-intervention score (ΔES reduction = pre − post). For within-group comparisons, mean changes, 95% confidence intervals, *t* values, *p* values, and Cohen’s *d_z_* were reported. For between-group comparisons of change scores, η^2^ was reported as the effect size (Lakens, 2013).

An exploratory multicategorical parallel mediation analysis was conducted using Model 4 of the PROCESS macro for SPSS (Hayes and Rockwood, 2017). Intervention condition was retained as a four-category predictor rather than being collapsed into a binary variable. Indicator coding was used, with traditional aerobic training designated as the reference condition. This coding generated three relative intervention contrasts: strength training versus traditional aerobic training, VR-based gamified exercise versus traditional aerobic training, and combined training versus traditional aerobic training. ΔSAS was entered as the dependent variable, whereas ΔCR and ΔES reduction were entered as parallel mediators. For statistical modeling purposes, ΔCR and ΔES reduction were entered in the mediator positions of the PROCESS model. However, because these variables and ΔSAS were calculated over the same pre–post interval, the term “mediator” refers only to their position in the statistical model and does not imply temporal precedence or causal mediation. For each intervention contrast, relative total effects, relative direct effects, mediator-specific relative indirect effects, and total relative indirect effects were estimated using 5,000 bootstrap samples. A relative indirect effect was considered statistically different from zero when its 95% bootstrap confidence interval did not include zero. Unstandardized effect estimates, standard errors, and 95% confidence intervals were reported. Exploratory bootstrap contrasts were additionally calculated to compare the magnitudes of the indirect effects across the three alternative exercise modalities. Because the sample size was not specifically powered for contrast-specific mediation and the mediators and outcome were represented by concurrent pre–post change scores, the analysis was interpreted as an exploratory assessment of relative indirect effects rather than as definitive evidence of temporally established or causal mediation.

## 3 Results

### 3.1 Assumption checks and data screening

Of the 85 randomized participants, 77 (90.6%) were included in the modified per-protocol complete-case analysis. Eight participants were excluded because of incomplete post-intervention data (*n* = 4), illness-related absence (*n* = 3), or university transfer (*n* = 1), with two exclusions in each group. No values were missing for the pre-test, post-test, or change scores of SAS-SV, cognitive reappraisal, or expressive suppression among the 77 participants included in the analysis.

The Shapiro–Wilk tests indicated that the pre-test and post-test scores were generally approximately normally distributed. All four groups met the normality assumption for the pre-test SAS-SV scores, with W values ranging from 0.929 to 0.977 and *p* values ranging from 0.147 to 0.895. For the post-test SAS-SV scores, only the traditional aerobic training group showed a significant deviation from normality, W = 0.897, *p* = 0.037, whereas the other three groups did not, W = 0.910–0.963, *p* = 0.073–0.641. Cognitive reappraisal scores did not significantly deviate from normality in any group at either pre-test, W = 0.908–0.978, *p* = 0.068–0.922, or post-test, W = 0.917–0.969, *p* = 0.087–0.755. Expressive suppression scores were also approximately normally distributed across all groups at pre-test, W = 0.929–0.971, *p* = 0.164–0.801, and post-test, W = 0.957–0.968, *p* = 0.511–0.727.

Several change scores, however, deviated significantly from normality. Changes in SAS-SV scores were non-normal in the traditional aerobic training group, W = 0.809, *p* = 0.001, the strength training group, W = 0.791, *p* < 0.001, and the VR-based gamified exercise group, W = 0.882, *p* = 0.024, but not in the combined training group, W = 0.909, *p* = 0.072. Cognitive reappraisal change scores deviated from normality in all four groups, W = 0.786–0.862, with *p* values ranging from < 0.001 to 0.011. Expressive suppression change scores also deviated from normality in all four groups, W = 0.737–0.883, with *p* values ranging from < 0.001 to 0.024.

The median-centered Levene tests supported homogeneity of variance for SAS-SV scores at pre-test and post-test and for SAS-SV change scores, *F*(3, 73) = 2.494, *p* = 0.067; *F*(3, 73) = 1.020, *p* = 0.389; and *F*(3, 73) = 0.843, *p* = 0.475, respectively. Homogeneity was also supported for cognitive reappraisal at pre-test, post-test, and for the change scores, *F*(3, 73) = 0.942, *p* = 0.425; *F*(3, 73) = 0.806, *p* = 0.495; and *F*(3, 73) = 1.622, *p* = 0.192, respectively. For expressive suppression, the homogeneity assumption was violated at pre-test, *F*(3, 73) = 3.922, *p* = 0.012, and post-test, *F*(3, 73) = 3.918, *p* = 0.012, but was satisfied for the change scores, *F*(3, 73) = 1.823, *p* = 0.150. Accordingly, Welch’s ANOVA was used for the cross-sectional comparisons of expressive suppression. The results showed no significant baseline group difference, Welch’s *F*(3, 37.90) = 2.121, *p* = 0.114, but a significant post-intervention group difference, Welch’s *F*(3, 38.48) = 6.050, *p* = 0.002.

Boxplot screening identified several mild outlying observations; however, none of the SAS-SV, cognitive reappraisal, or expressive suppression scores at pre-test, post-test, or for change exceeded the prespecified extreme-outlier criterion of |Z| > 3.29. All flagged observations remained within the theoretical ranges of the corresponding scales and were retained. Because the within-subject factor time consisted of only two levels, the assumption of sphericity was satisfied by definition (*ε* = 1.000), and no sphericity correction was required.

Kruskal–Wallis tests showed significant overall between-group differences in SAS-SV change scores, H(3) = 51.528, *p* < 0.001, *ε*^2^ = 0.665; cognitive reappraisal, H(3) = 35.381, *p* < 0.001, *ε*^2^ = 0.444; and expressive suppression, H(3) = 33.222, *p* < 0.001, *ε*^2^ = 0.414. Wilcoxon signed-rank tests also confirmed significant pre–post changes in SAS-SV scores and cognitive reappraisal in all four groups, all *p* < 0.001. Expressive suppression decreased significantly in the traditional aerobic training group, *p* = 0.002, and in the other three groups, all *p* < 0.001. These sensitivity analyses were consistent with the overall omnibus conclusions of the parametric analyses.

### 3.2 Participant characteristics

No statistically significant baseline differences were observed among the four groups in SAS-SV scores, *F*(3, 73) = 0.270, *p* = 0.847, or cognitive reappraisal, *F*(3, 73) = 0.578, *p* = 0.632. Because the homogeneity-of-variance assumption was violated for expressive suppression at baseline, Welch’s ANOVA was used and showed no significant baseline group difference, Welch’s *F*(3, 37.90) = 2.121, *p* = 0.114. Similarly, no significant between-group differences were found in age, height, body weight, or BMI, all *p* > 0.05. The four groups were generally comparable at baseline (Table 2).

**Table 2:** Baseline characteristics of the participants.

Variable	Total (*N* = 77)	Traditional aerobic training (*n* = 20)	Strength training group (*n* = 19)	VR-based gamified exercise (*n* = 19)	Combined training group (*n* = 19)	*P*
Age (years)	19.49 ± 1.44	19.85 ± 0.99	19.21 ± 1.13	19.26 ± 1.69	19.63 ± 1.80	>0.05
Height (cm)	175.22 ± 6.07	175.20 ± 4.76	177.16 ± 7.60	174.58 ± 6.63	173.95 ± 4.88	
Body weight (kg)	66.23 ± 11.12	69.03 ± 9.20	64.68 ± 9.64	63.08 ± 13.60	68.00 ± 11.39	
BMI (kg/m^2^)	21.56 ± 3.43	22.55 ± 3.47	20.64 ± 3.06	20.55 ± 3.39	22.46 ± 3.51	
Attendance rate	88.8%	88.2%	92.2%	87.1%	87.6%	

Data are presented as mean ± SD. Between-group differences in age, height, body weight, and BMI were assessed using one-way analysis of variance. BMI, body mass index.

Regarding intervention adherence, the overall mean attendance rate among the 77 participants included in the final analysis was 88.8%. The group-specific attendance rates were 88.2% for traditional aerobic training, 92.2% for strength training, 87.1% for VR-based gamified exercise, and 87.6% for combined training. All participants included in the final analysis met the pre-specified attendance criterion of at least 80%. No injuries or other adverse events were documented during the 12-week intervention. Attendance and adverse-event records were the only intervention-fidelity indicators retained in a sufficiently complete and standardized format for quantitative reporting. Although heart-rate responses, Borg CR-10 ratings, time within the target heart-rate zone, and training-completion indicators were monitored during intervention delivery, the corresponding session-level records were not sufficiently complete and standardized for group-level quantitative analysis. Therefore, achieved exercise-intensity and exercise-dose comparability across the four intervention groups could not be quantitatively verified.

### 3.3 Pre–post changes in SAS-SV scores across intervention groups

**Figure 2:**
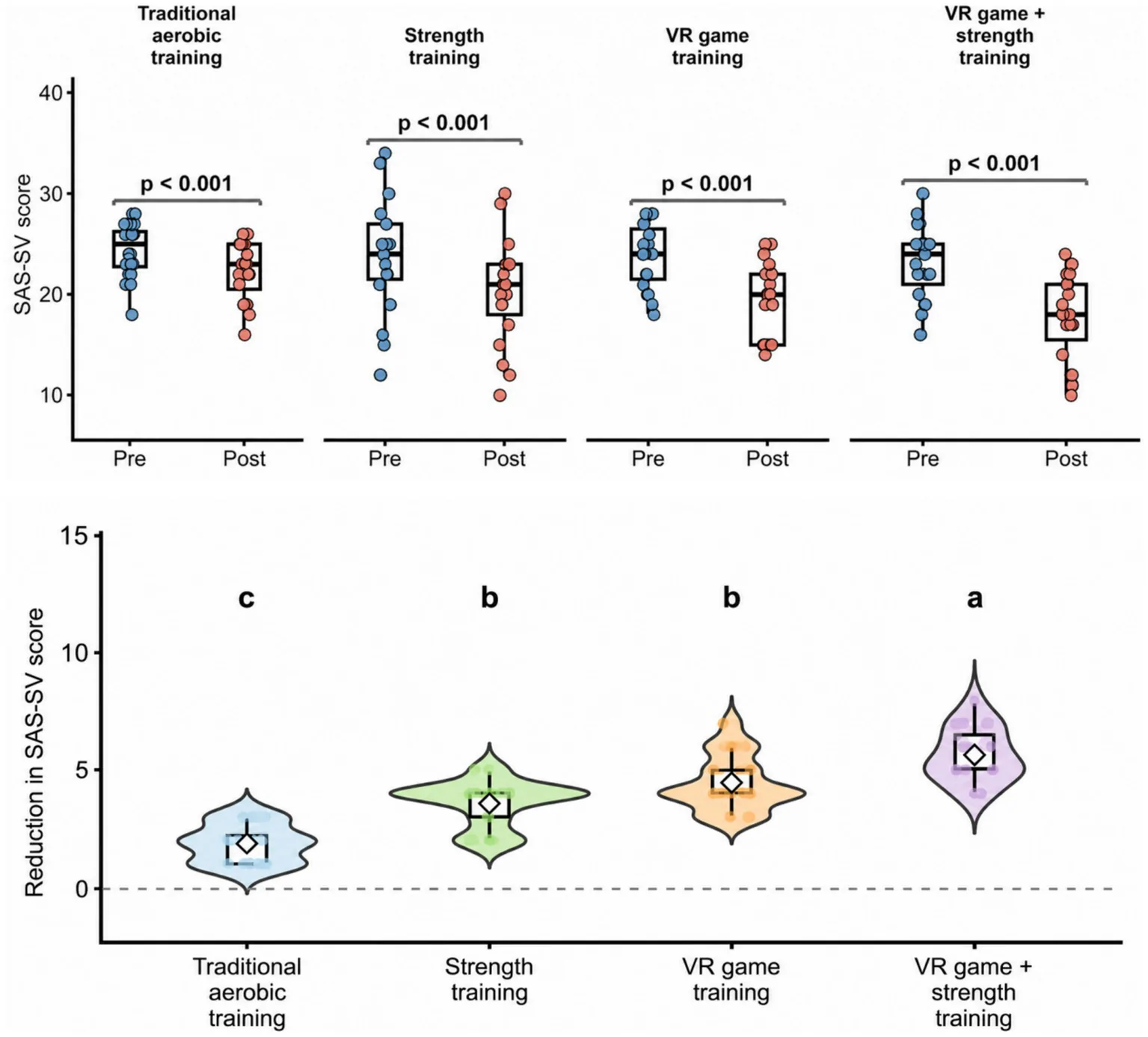
Changes in SAS-SV scores from pre to post-intervention and group differences in score reduction. Top: Within-group pre–post changes. ***p < 0.001. Bottom: Between-group reductions in SAS-SV scores. Different letters indicate significant differences (*p* < 0.05).

As shown in Figure 2, the mixed-design ANOVA showed a significant main effect of time on SAS-SV scores, *F*(1, 73) = 1079.274, *p* < 0.001, partial η^2^ = 0.937, whereas the main effect of group was not significant, *F*(3, 73) = 1.884, *p* = 0.140, partial η^2^ = 0.072. More importantly, the time × group interaction was significant, *F*(3, 73) = 45.205, *p* < 0.001, partial η^2^ = 0.650, indicating that the magnitude of the pre–post reduction in SAS-SV scores differed among the four intervention groups. Before the intervention, there was no statistically significant difference in SAS-SV scores among the four groups, indicating that the baseline levels were comparable across groups, *F*(3, 73) = 0.270, *p* = 0.847. After the 12-week intervention, SAS-SV scores decreased significantly in all four groups. In the traditional aerobic training group, scores decreased from 24.40 ± 2.72 to 22.50 ± 2.96, corresponding to a mean reduction of 1.90 ± 0.79 points [95% CI (1.53, 2.27), Cohen’s *d_z_* = 2.41], *t*(19) = 10.78, *p* < 0.001. In the strength training group, scores decreased from 23.74 ± 5.70 to 20.16 ± 5.21, corresponding to a mean reduction of 3.58 ± 0.96 points [95% CI (3.12, 4.04), Cohen’s *d_z_* = 3.72], *t*(18) = 16.23, *p* < 0.001. In the VR-based gamified exercise group, scores decreased from 23.79 ± 3.17 to 19.37 ± 3.65, corresponding to a mean reduction of 4.42 ± 1.17 points [95% CI (3.86, 4.98), Cohen’s *d_z_* = 3.78], *t*(18) = 16.47, *p* < 0.001. In the combined training group, scores decreased from 23.26 ± 3.68 to 17.63 ± 4.21, corresponding to a mean reduction of 5.63 ± 1.16 points [95% CI (5.07, 6.19), Cohen’s *d_z_* = 4.83], *t*(18) = 21.07, *p* < 0.001.

Between-group comparisons showed significant differences in post-intervention SAS-SV scores among the four groups, *F*(3, 73) = 4.798, *p* = 0.004. Follow-up comparisons of SAS-SV reduction scores were therefore conducted using Bonferroni-adjusted pairwise tests. The combined training group showed a significantly larger reduction than the traditional aerobic training group, strength training group, and VR-based gamified exercise group. Both the strength training group and the VR-based gamified exercise group showed significantly larger reductions than the traditional aerobic training group, whereas the difference between the strength training group and the VR-based gamified exercise group was not significant, *p* = 0.123. Overall, the magnitude of the pre–post reduction in SAS-SV scores followed the pattern: combined training > VR-based gamified exercise ≈ strength training > traditional aerobic training. This pattern reflects relative differences among the four active intervention conditions and does not establish the absolute effect of exercise compared with no intervention.

### 3.4 Pre–post changes in cognitive reappraisal across intervention groups

**Figure 3:**
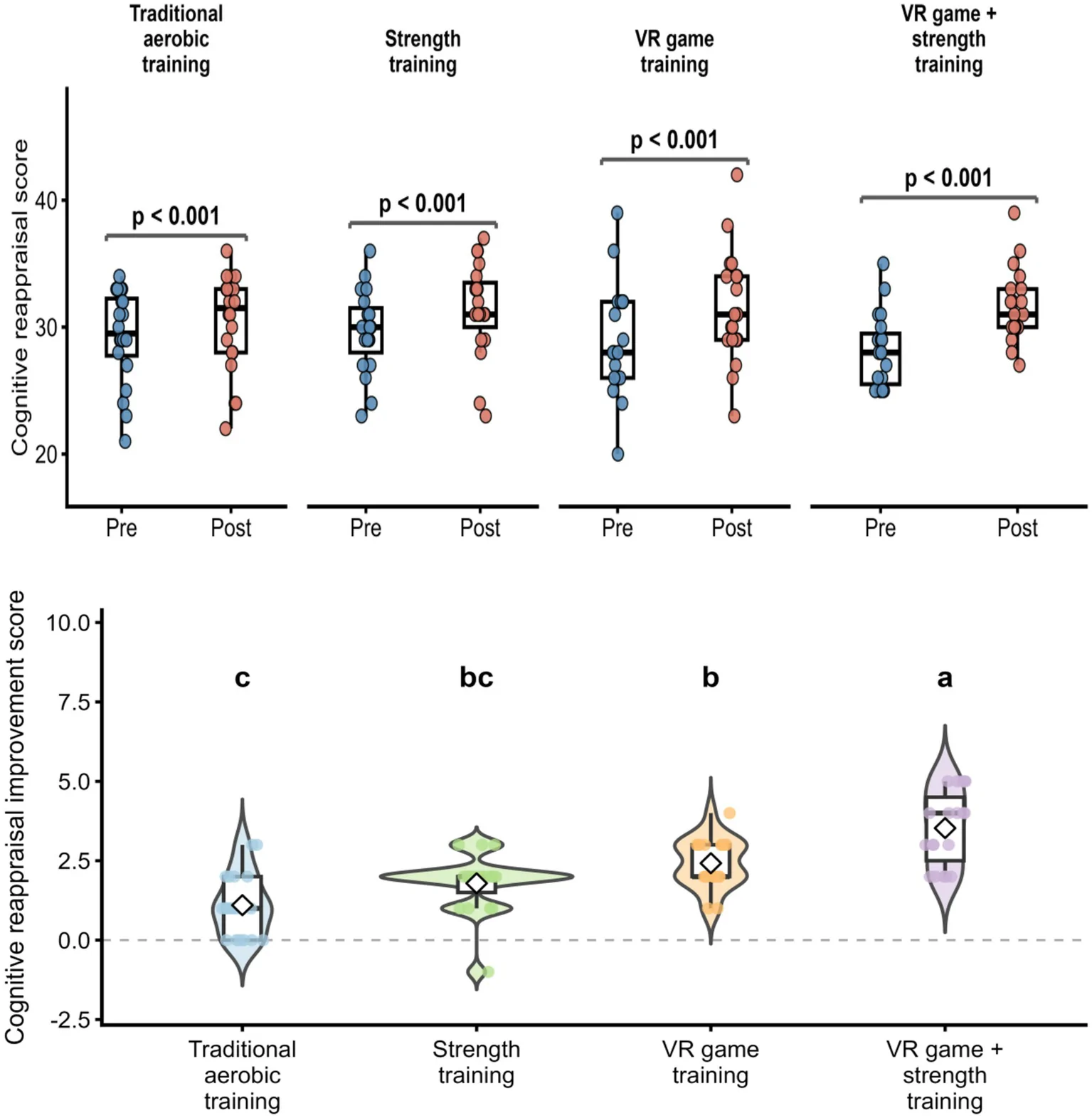
Changes in cognitive reappraisal scores from pre to post-intervention and group differences in cognitive reappraisal change. Top: Within-group pre–post changes. ***p < 0.001. Bottom: Between-group changes in cognitive reappraisal scores. Different letters indicate significant between-group differences at *p* < 0.05.

As shown in Figure 3, the mixed-design ANOVA showed a significant main effect of time on cognitive reappraisal, *F*(1, 73) = 396.294, *p* < 0.001, partial η^2^ = 0.844, whereas the main effect of group was not significant, *F*(3, 73) = 0.149, *p* = 0.930, partial η^2^ = 0.006. The time × group interaction was significant, *F*(3, 73) = 21.992, *p* < 0.001, partial η^2^ = 0.475, indicating that the magnitude of the pre–post increase in cognitive reappraisal differed among the four groups. Before the intervention, there was no statistically significant difference in cognitive reappraisal scores among the four groups, *F*(3, 73) = 0.578, *p* = 0.632, indicating that baseline cognitive reappraisal levels were comparable across groups. After the 12-week intervention, cognitive reappraisal scores increased significantly within all four groups. In the traditional aerobic training group, scores increased from 29.30 ± 3.70 to 30.40 ± 3.84, corresponding to a mean increase of 1.10 ± 0.97 points [95% CI (0.65, 1.55), Cohen’s *d_z_* = 1.14], *t*(19) = 5.08, *p* < 0.001. In the strength training group, scores increased from 29.63 ± 3.30 to 31.42 ± 3.73, corresponding to a mean increase of 1.79 ± 0.92 points [95% CI (1.35, 2.23), Cohen’s *d_z_* = 1.95], *t*(18) = 8.50, *p* < 0.001. In the VR-based gamified exercise group, scores increased from 29.11 ± 4.43 to 31.53 ± 4.41, corresponding to a mean increase of 2.42 ± 0.77 points [95% CI (2.05, 2.79), Cohen’s *d_z_* = 3.15], *t*(18) = 13.73, *p* < 0.001. In the combined training group, scores increased from 28.16 ± 2.91 to 31.68 ± 2.89, corresponding to a mean increase of 3.53 ± 1.17 points [95% CI (2.96, 4.09) Cohen’s *d_z_* = 3.01], *t*(18) = 13.11, *p* < 0.001.

Between-group comparisons showed no significant difference in post-intervention cognitive reappraisal scores among the four groups, *F*(3, 73) = 0.472, *p* = 0.702. Given the significant time × group interaction, cognitive reappraisal change scores were subsequently compared using Bonferroni-adjusted pairwise tests. The combined training group showed a significantly larger increase than the traditional aerobic training group, strength training group, and VR-based gamified exercise group. The VR-based gamified exercise group showed a significantly larger increase than the traditional aerobic training group, *p* < 0.001. However, the difference between the strength training group and the traditional aerobic training group was not significant, *p* = 0.171, nor was the difference between the strength training group and the VR-based gamified exercise group, *p* = 0.164. Overall, the combined training group showed the largest pre–post increase in cognitive reappraisal. This finding indicates a larger relative change under the combined condition but does not establish that the intervention itself activated adaptive emotion-regulation processes.

### 3.5 Pre–post changes in expressive suppression across intervention groups

**Figure 4:**
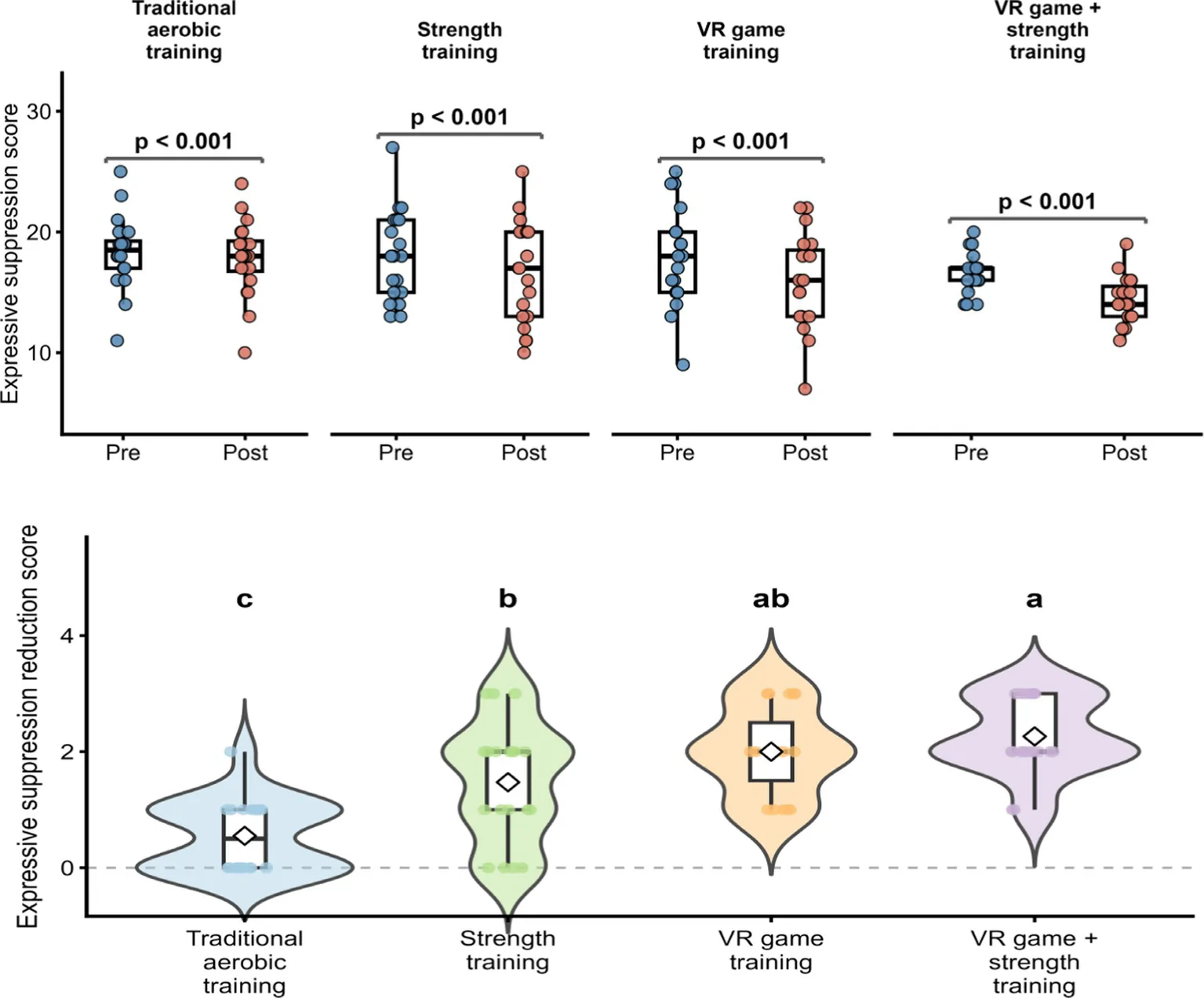
Changes in expressive suppression scores from pre to post-intervention and group differences in expressive suppression reduction. Top: Within-group pre–post changes; *p* = 0.001 for traditional aerobic training and *p* < 0.001 for the other three groups. Bottom: Between-group differences in expressive suppression reduction scores. Different letters indicate significant between-group differences at *p* < 0.05.

As shown in Figure 4, the mixed-design ANOVA showed a significant main effect of time on expressive suppression, *F*(1, 73) = 314.771, *p* < 0.001, partial η^2^ = 0.812, whereas the main effect of group was not significant, *F*(3, 73) = 2.054, *p* = 0.114, partial η^2^ = 0.078. The time × group interaction was significant, *F*(3, 73) = 18.860, *p* < 0.001, partial η^2^ = 0.437, indicating that the magnitude of reduction in expressive suppression differed among the four intervention groups. Because the homogeneity-of-variance assumption was violated at baseline, Welch’s ANOVA was used and indicated no significant baseline group difference in expressive suppression, Welch’s *F*(3, 37.90) = 2.121, *p* = 0.114, indicating that baseline expressive suppression levels were comparable across groups. After the 12-week intervention, expressive suppression scores decreased significantly within all four groups. In the traditional aerobic training group, scores decreased from 18.35 ± 2.98 to 17.80 ± 3.12, corresponding to a mean reduction of 0.55 ± 0.60 points [95% CI (0.27, 0.83), Cohen’s *d_z_* = 0.91], *t*(19) = 4.07, *p* = 0.001. In the strength training group, scores decreased from 18.05 ± 3.75 to 16.58 ± 4.29, corresponding to a mean reduction of 1.47 ± 1.02 points [95% CI (0.98, 1.97), Cohen’s *d_z_* = 1.44], *t*(18) = 6.30, *p* < 0.001. In the VR-based gamified exercise group, scores decreased from 17.84 ± 4.11 to 15.84 ± 3.91, corresponding to a mean reduction of 2.00 ± 0.75 points [95% CI (1.64, 2.36), Cohen’s *d_z_* = 2.68], *t*(18) = 11.70, *p* < 0.001. In the combined training group, scores decreased from 16.63 ± 1.67 to 14.37 ± 1.92, corresponding to a mean reduction of 2.26 ± 0.65 points [95% CI (1.95, 2.58), Cohen’s *d_z_* = 3.46], *t*(18) = 15.10, *p* < 0.001.

Because the homogeneity-of-variance assumption was violated for the post-intervention expressive suppression scores, Welch’s ANOVA was used. The results showed a significant post-intervention difference among the four groups, Welch’s *F*(3, 38.48) = 6.050, *p* = 0.002. Comparisons of expressive suppression reduction scores also showed significant between-group differences, *F*(3, 73) = 18.860, *p* < 0.001, η^2^ = 0.437. Bonferroni-adjusted comparisons showed that the strength training group, VR-based gamified exercise group, and combined training group all had significantly larger reductions than the traditional aerobic training group. The combined training group also showed a significantly larger reduction than the strength training group, *p* = 0.044. However, the difference between the VR-based gamified exercise group and the strength training group was not significant, *p* = 0.467, nor was the difference between the VR-based gamified exercise group and the combined training group, *p* = 1.000. Overall, the combined training group showed the largest pre–post reduction in expressive suppression, followed by the VR-based gamified exercise group. This pattern reflects relative differences among the four active conditions and does not establish that exercise itself reduced participants’ reliance on expressive suppression. Within-group mean changes, 95% confidence intervals, and Cohen’s *d_z_* effect sizes for all three outcomes are summarized in Table 3.

**Table 3:** Within-group changes and effect sizes across intervention groups.

Outcome	Group	Mean change ± SD	95% CI	Cohen’s *d_z_*
ΔSAS	Traditional aerobic training	1.90 ± 0.79	[1.53, 2.27]	2.41
	Strength training	3.58 ± 0.96	[3.12, 4.04]	3.72
	VR-based gamified exercise	4.42 ± 1.17	[3.86, 4.98]	3.78
	Combined training	5.63 ± 1.16	[5.07, 6.19]	4.83
ΔCR	Traditional aerobic training	1.10 ± 0.97	[0.65, 1.55]	1.14
	Strength training	1.79 ± 0.92	[1.35, 2.23]	1.95
	VR-based gamified exercise	2.42 ± 0.77	[2.05, 2.79]	3.15
	Combined training	3.53 ± 1.17	[2.96, 4.09]	3.01
ΔES reduction	Traditional aerobic training	0.55 ± 0.60	[0.27, 0.83]	0.91
	Strength training	1.47 ± 1.02	[0.98, 1.97]	1.44
	VR-based gamified exercise	2.00 ± 0.75	[1.64, 2.36]	2.68
	Combined training	2.26 ± 0.65	[1.95, 2.58]	3.46

Positive change scores indicate improvement. ΔSAS was calculated as the pre-intervention SAS-SV total score minus the post-intervention SAS-SV total score, with higher values indicating a larger reduction in problematic smartphone use tendencies; ΔCR was calculated as the post-intervention score minus the pre-intervention score; and ΔES reduction was calculated as the pre-intervention score minus the post-intervention score. The 95% confidence intervals refer to the mean change scores. Cohen’s d_z_ was calculated as the mean change divided by the standard deviation of the change score.

### 3.6 Exploratory multicategorical analysis of relative indirect effects

**Figure 5:**
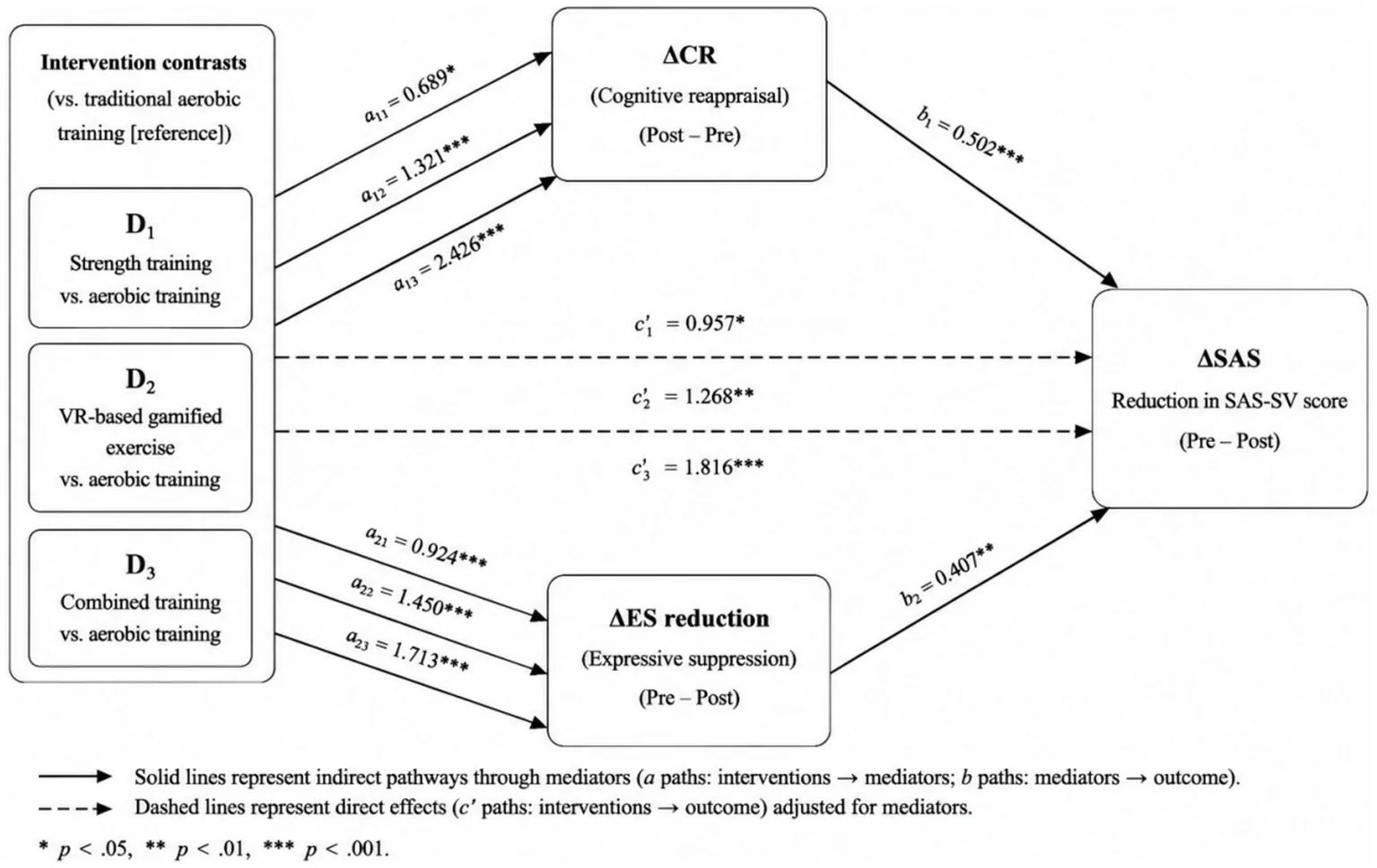
Exploratory multicategorical relative indirect-effect model with traditional aerobic training as the reference condition. Intervention condition was indicator-coded with traditional aerobic training as the reference category. *D1* represents strength training versus traditional aerobic training; *D2* represents VR-based gamified exercise versus traditional aerobic training; and *D3* represents combined training versus traditional aerobic training. *ΔCR* and *ΔES* reduction were entered as parallel mediators, and *ΔSAS* was entered as the outcome. The arrows represent regression-based statistical associations among concurrent pre–post change scores and should not be interpreted as evidence of temporal ordering or causal mediation.

An exploratory multicategorical analysis of relative indirect effects was conducted to examine the statistical extent to which differences in cognitive reappraisal change and expressive suppression reduction were associated with the relative intervention contrasts in SAS-SV score reduction. Traditional aerobic training was designated as the reference condition, generating three separate intervention contrasts: strength training versus traditional aerobic training, VR-based gamified exercise versus traditional aerobic training, and combined training versus traditional aerobic training. The contrast-specific estimates are presented in Table 4 and Figure 5.

**Table 4:** Exploratory multicategorical relative indirect-effect estimates.

Intervention contrast	Effect	Estimate	SE/BootSE	95% CI
Strength training vs traditional aerobic training	Relative total effect	1.679	0.330	[1.021, 2.337]
	Relative direct effect	0.957	0.301	[0.358, 1.556]
	Relative indirect effect via ΔCR	0.346	0.176	[0.044, 0.729]
	Relative indirect effect via ΔES reduction	0.376	0.176	[0.104, 0.787]
	Total relative indirect effect	0.722	0.258	[0.296, 1.305]
VR-based gamified exercise vs traditional aerobic training	Relative total effect	2.521	0.330	[1.863, 3.179]
	Relative direct effect	1.268	0.347	[0.575, 1.961]
	Relative indirect effect via ΔCR	0.664	0.178	[0.338, 1.030]
	Relative indirect effect via ΔES reduction	0.590	0.214	[0.216, 1.046]
	Total relative indirect effect	1.253	0.296	[0.721, 1.868]
Combined training vs traditional aerobic training	Relative total effect	3.732	0.330	[3.074, 4.389]
	Relative direct effect	1.816	0.414	[0.990, 2.642]
	Relative indirect effect via ΔCR	1.219	0.293	[0.692, 1.831]
	Relative indirect effect via ΔES reduction	0.697	0.226	[0.279, 1.158]
	Total relative indirect effect	1.916	0.382	[1.219, 2.712]

*N* = 77. Traditional aerobic training was designated as the reference condition. Intervention condition was represented using indicator coding, generating separate contrasts for strength training, VR-based gamified exercise, and combined training relative to traditional aerobic training. Effects are unstandardized. Indirect effects were estimated using 5,000 bootstrap samples. ΔSAS, pre-intervention minus post-intervention SAS-SV total score; higher values indicate a larger reduction in problematic smartphone use tendencies; ΔCR, post-intervention minus pre-intervention cognitive reappraisal score; ΔES reduction, pre-intervention minus post-intervention expressive suppression score. Higher values indicate greater improvement.

Intervention condition was statistically associated with between-group differences in cognitive reappraisal improvement, *R*^2^ = 0.475, *F*(3, 73) = 21.992, *p* < 0.001, and reduction in expressive suppression, *R*^2^ = 0.437, *F*(3, 73) = 18.860, *p* < 0.001. Relative to traditional aerobic training, strength training (*B* = 0.689, SE = 0.310, *p* = 0.029), VR-based gamified exercise (*B* = 1.321, SE = 0.310, *p* < 0.001), and combined training (*B* = 2.426, SE = 0.310, *p* < 0.001) showed greater improvements in cognitive reappraisal. The corresponding relative effects on expressive suppression reduction were 0.924 (SE = 0.247, *p* < 0.001), 1.450 (SE = 0.247, *p* < 0.001), and 1.713 (SE = 0.247, *p* < 0.001), respectively. In the outcome model, both improvement in cognitive reappraisal (*B* = 0.502, SE = 0.103, *p* < 0.001) and reduction in expressive suppression (*B* = 0.407, SE = 0.129, *p* = 0.002) were positively associated with larger reductions in SAS-SV scores after controlling for intervention condition and the other mediator.

Relative to traditional aerobic training, strength training showed a relative total effect of 1.679 on SAS-SV score reduction [SE = 0.330, 95% CI (1.021, 2.337)]. After the two mediators were included, the relative direct effect remained significant [effect = 0.957, SE = 0.301, 95% CI (0.358, 1.556)]. The relative indirect effect through cognitive reappraisal was 0.346 [BootSE = 0.176, 95% bootstrap CI (0.044, 0.729)], and the relative indirect effect through expressive suppression reduction was 0.376 [BootSE = 0.176, 95% bootstrap CI (0.104, 0.787)]. The total relative indirect effect was 0.722 [BootSE = 0.258, 95% bootstrap CI (0.296, 1.305)].

Relative to traditional aerobic training, VR-based gamified exercise showed a relative total effect of 2.521 [SE = 0.330, 95% CI (1.863, 3.179)] and a relative direct effect of 1.268 [SE = 0.347, 95% CI (0.575, 1.961)]. The relative indirect effect through cognitive reappraisal was 0.664 [BootSE = 0.178, 95% bootstrap CI (0.338, 1.030)], whereas the relative indirect effect through expressive suppression reduction was 0.590 [BootSE = 0.214, 95% bootstrap CI (0.216, 1.046)]. The total relative indirect effect was 1.253 [BootSE = 0.296, 95% bootstrap CI (0.721, 1.868)].

Relative to traditional aerobic training, combined training showed a relative total effect of 3.732 [SE = 0.330, 95% CI (3.074, 4.389)] and a relative direct effect of 1.816 [SE = 0.414, 95% CI (0.990, 2.642)]. The relative indirect effect through cognitive reappraisal was 1.219 [BootSE = 0.293, 95% bootstrap CI (0.692, 1.831)], and the relative indirect effect through expressive suppression reduction was 0.697 [BootSE = 0.226, 95% bootstrap CI (0.279, 1.158)]. The total relative indirect effect was 1.916 [BootSE = 0.382, 95% bootstrap CI (1.219, 2.712)].

The 95% bootstrap confidence intervals for the mediator-specific relative indirect effects excluded zero for all three contrasts, while the corresponding relative direct effects also remained different from zero. Thus, the model yielded both relative indirect and relative direct components; this pattern should not be interpreted as evidence of partial causal mediation.

Exploratory bootstrap comparisons showed that the total relative indirect effect was larger for VR-based gamified exercise than for strength training [difference = 0.531, BootSE = 0.204, 95% bootstrap CI (0.164, 0.957)], larger for combined training than for strength training [difference = 1.194, BootSE = 0.279, 95% bootstrap CI (0.670, 1.759)], and larger for combined training than for VR-based gamified exercise [difference = 0.662, BootSE = 0.225, 95% bootstrap CI (0.241, 1.127)]. These exploratory comparisons suggest that variation in the total indirect effect across modalities was primarily associated with the cognitive reappraisal pathway. Because these pairwise comparisons were exploratory and were not adjusted for multiple testing, they should be interpreted cautiously.

## 4 Discussion

This study identified modality-related differences in pre–post changes in SAS-SV scores and emotion regulation, together with exploratory differences in the corresponding relative indirect-effect components.

### 4.1 Comparative changes in problematic smartphone use tendencies across exercise modalities

SAS-SV scores decreased from pre to post-intervention in all four active conditions, with the magnitude of reduction differing across modalities. The combined training group showed the largest reduction, followed by the VR-based gamified exercise and strength training groups, whereas the traditional aerobic training group showed the smallest reduction. Because no non-exercise, wait-list, or attention-control condition was included, these within-group changes cannot be attributed specifically to exercise and may also reflect repeated measurement, participant expectations, researcher attention, social interaction, or natural changes over time. Thus, the randomized design supports comparisons of relative pre–post changes among the four active modalities but does not establish the absolute effect of exercise per se.

The large effect-size estimates should also be interpreted cautiously. The substantial time effects and time × group interaction reflect changes within the present repeated-measures model rather than exercise-versus-no-exercise effects. Similarly, Cohen’s *d_z_* values of 2.41–4.83 were calculated using the standard deviation of individual change scores and therefore indicate highly consistent within-group changes rather than equivalently large between-group treatment effects. The small and relatively homogeneous sample, modified per-protocol analysis, repeated self-report assessment, and limited variability in change scores may also have contributed to the magnitude of these estimates. Replication using larger samples, intention-to-treat analyses, objective smartphone-use indicators, and appropriate control conditions is therefore needed.

The I-PACE model provides a theoretical framework for interpreting the observed modality-related differences because it emphasizes the roles of affective responses, cognitive processes, reward experience, and executive control in problematic digital-media use (Brand et al., 2025). Strength training provides structured goals, progressive loading, and performance feedback, whereas VR-based gamified exercise adds immersive scenarios, immediate feedback, and game-based rewards. The combination of these features is theoretically compatible with the larger reduction observed in the combined training group. However, variables such as executive function, craving, reward processing, self-control, and exercise motivation were not directly measured. Moreover, changes in cognitive reappraisal, expressive suppression, and SAS-SV scores were assessed over the same pre–post interval. Accordingly, the findings indicate concurrent statistical associations but do not establish that these proposed mechanisms caused the reductions in SAS-SV scores or preceded them temporally.

### 4.2 Comparative changes in cognitive reappraisal across exercise modalities

Cognitive reappraisal scores increased from pre to post-intervention in all four active conditions, with the magnitude of increase differing across modalities. The combined training group showed the largest increase, followed by the VR-based gamified exercise group, whereas smaller increases were observed in the strength training and traditional aerobic training groups. These findings indicate relative differences in pre–post change among the four active intervention conditions.

Cognitive reappraisal involves modifying the interpretation of an emotion-eliciting situation before the emotional response is fully developed and is generally considered a more adaptive emotion regulation strategy than expressive suppression (Gross, 1998; Gross and John, 2003). Exercise contexts may provide opportunities to reinterpret fatigue, challenge, task failure, and goal attainment, which is theoretically consistent with the observed increases in cognitive reappraisal. Previous research has also suggested that exercise may support stress recovery and emotion regulation processes (Bernstein and McNally, 2018; Tao et al., 2025). The larger increases observed in the VR-based gamified exercise and combined training groups may be compatible with their greater task variation, immediate feedback, cognitive engagement, and opportunities for goal attainment.

However, these moment-to-moment cognitive processes were not directly measured, and the absence of a non-exercise or attention-control condition prevents conclusions about whether exercise itself caused the observed increases. Accordingly, the findings should be interpreted as relative between-modality differences in cognitive reappraisal change rather than evidence that the interventions activated specific cognitive-change mechanisms.

### 4.3 Comparative changes in expressive suppression across exercise modalities

Expressive suppression scores decreased from pre to post-intervention in all four active conditions, with the magnitude of reduction differing across modalities. The combined training group showed the largest reduction, followed by the VR-based gamified exercise group, whereas the traditional aerobic training group showed the smallest reduction. These findings indicate relative differences in pre-post change among the four active intervention conditions. However, because no non-exercise, wait-list, or attention-control condition was included, the reductions cannot be attributed specifically to physical exercise.

The observed reductions are theoretically compatible with the possibility that exercise contexts provide opportunities for bodily engagement, emotional recovery, and emotional expression. Physical activity has been associated with reductions in anxiety, depression, and psychological stress, and may buffer the adverse effects of emotion-regulation difficulties on stress recovery (Biddle and Asare, 2011; Bernstein and McNally, 2018). Traditional aerobic training may facilitate stress relief through sustained bodily activity (Mizzi et al., 2022), but its relatively limited interactivity, feedback, and contextual variation may be compatible with the smaller reduction in expressive suppression observed in this group. By contrast, VR-based gamified exercise provides virtual scenarios, task challenges, immediate feedback, and game-based rewards (Ochi et al., 2022). These features may allow participants to experience emotions such as excitement, frustration, achievement, and pleasure and to externalize some of these experiences through motor responses, task completion, and interactive feedback. The larger reduction observed in the VR-based gamified exercise group may therefore be associated with its greater interactivity and contextual variability, although these processes were not directly measured.

The combined training group showed the most pronounced reduction in expressive suppression. This pattern is theoretically compatible with the complementary presence of immersive emotional engagement and structured goal attainment. VR-based gamified exercise may provide opportunities for interactive emotional experience, whereas strength training offers clear goals, progressive overload, and feedback on physical capability, which may support perceived control and task mastery (Barbour et al., 2024; Martinez Kercher et al., 2024). For example, participants may experience frustration when failing to complete a VR task, achievement after improving performance, or increased control when successfully completing progressively demanding strength exercises. Such experiences may provide opportunities to respond to emotions through bodily action, task adjustment, and goal completion rather than relying solely on outward emotional suppression. Nevertheless, the present findings do not establish that these intervention features caused the larger reduction observed in the combined group.

This study assessed only participants’ general tendency to suppress outward emotional expression and did not directly measure emotional-expression behaviors, physiological responses, perceived control, or moment-to-moment emotional experiences. Therefore, the observed pattern is compatible with, but does not verify, the proposed processes of emotional expression, bodily engagement, and perceived control. Future studies should incorporate behavioral observations, physiological indicators, ecological momentary assessments, and repeated measures of emotional experience to examine whether these processes temporally precede subsequent changes in expressive suppression.

### 4.4 Exploratory relative indirect-effect patterns involving emotion regulation

The multicategorical analysis estimated separate relative indirect effects for strength training, VR-based gamified exercise, and combined training compared with traditional aerobic training. For all three contrasts, the 95% bootstrap confidence intervals for the relative indirect effects involving cognitive reappraisal improvement and expressive suppression reduction excluded zero. The total relative indirect-effect component was largest for combined training, followed by VR-based gamified exercise and strength training. Exploratory pairwise comparisons further indicated that between-modality differences were more evident for the cognitive reappraisal component than for the expressive suppression component. These findings describe differences in the statistical decomposition of the intervention contrasts but do not demonstrate that the exercise modalities operated through distinct emotion-regulation mechanisms.

The relative direct effects remained different from zero after changes in cognitive reappraisal and expressive suppression were included in the model. Thus, these concurrent emotion-regulation changes did not statistically account for the full relative differences in SAS-SV score reduction. Other unmeasured factors may also have contributed to the remaining contrasts, including temporary displacement of smartphone use, sleep quality, peer interaction, perceived competence, exercise enjoyment, and self-control. However, these variables were not assessed and should be regarded as possible explanations rather than established mechanisms.

Because cognitive reappraisal, expressive suppression, and SAS-SV scores were measured over the same pre–post interval, temporal precedence could not be established. Reductions in SAS-SV scores may have preceded emotion-regulation changes, emotion-regulation changes may have preceded SAS-SV score reductions, or both may have changed concurrently. Accordingly, the relative indirect effects should be interpreted as exploratory statistical associations rather than evidence of causal mediation. Future studies should assess emotion regulation repeatedly during the intervention and measure SAS-SV outcomes at later follow-up points to examine temporal ordering.

### 4.5 Implications

First, from the perspective of comparative intervention outcomes, the combined program showed the largest pre–post reduction in SAS-SV scores and the largest changes in cognitive reappraisal and expressive suppression among the four active conditions. Because the participants were not selected using diagnostic criteria or a screening cutoff, these implications concern general college students with varying problematic smartphone use tendencies rather than the treatment of clinically diagnosed smartphone addiction.

Second, in terms of implementation feasibility, the combined program may be suitable for preliminary feasibility testing in university settings. However, its broader implementation requires further evaluation of cost, equipment accessibility, instructor training, participant acceptability, and scalability. On the one hand, VR-based gamified exercise is characterized by enjoyment, immersion, and immediate feedback, which may enhance college students’ intrinsic motivation to participate in physical activity; on the other hand, strength training has clear goals, adjustable loads, and can be adapted to different exercise settings, making it suitable for integration into university physical education courses, extracurricular exercise programs, and mental health activities.

Third, from the perspective of future mechanism-focused research, the exploratory analysis showed that the magnitudes of the relative indirect effects involving cognitive reappraisal and expressive suppression differed across intervention contrasts. These findings support assessing emotion-regulation outcomes separately in future comparisons of exercise modalities, but they do not demonstrate that the modalities operated through distinct mechanisms. Confirmation requires repeated mediator assessments during the intervention and later measurement of SAS-SV outcomes.

Fourth, from the perspective of policy and educational practice, universities may consider pilot testing and formally evaluating such exercise programs within physical education courses, extracurricular activities, or student mental-health initiatives. However, implementation should remain exploratory until studies with appropriate non-exercise or attention-control conditions have established whether exercise provides benefits beyond expectancy, researcher attention, social interaction, and natural changes over time.

Fifth, from a theoretical perspective, this study discussed potential mechanisms such as executive function, self-regulation, exercise motivation, and reward processing; however, the research design did not directly measure these variables. Future studies should include repeated assessments of executive function, self-regulation, exercise motivation, and reward processing to examine whether changes in these variables temporally precede subsequent changes in SAS-SV scores.

### 4.6 Strengths and limitations

First, although the sample size was sufficient for the primary repeated-measures analyses, the group-specific samples remained relatively small for the exploratory multicategorical mediation analysis. The primary analyses were based on a modified per-protocol complete-case sample of 77 participants rather than the intention-to-treat population of all 85 randomized participants. Although post-randomization exclusions were numerically balanced across the four groups, excluding participants after randomization may have introduced attrition or selection bias, weakened the protection afforded by random allocation, and limited the applicability of the findings to all randomized participants. The small and relatively homogeneous single-institution sample may also have limited the precision and stability of the estimates.

Second, cognitive reappraisal, expressive suppression, and SAS-SV scores were assessed at the same two measurement occasions, and the analysis was based on concurrent pre–post change scores. Consequently, the temporal ordering of emotion-regulation changes and SAS-SV score reductions could not be established. The relative indirect effects should therefore be interpreted as statistical associations rather than causal mediation. Future studies should assess emotion regulation repeatedly during the intervention and measure SAS-SV outcomes at later time points.

Third, this study did not include a non-exercise, wait-list, or attention-control condition. Therefore, the observed pre–post changes cannot be attributed specifically to exercise and may also reflect repeated measurement, participant expectations, researcher attention, social interaction, or natural changes over time. In addition, no long-term follow-up assessment was conducted, preventing conclusions about the persistence of the observed changes. Future trials should include appropriate control conditions and follow-up assessments to determine whether the changes exceed nonspecific intervention effects and are maintained over time.

Fourth, participants were not recruited using diagnostic criteria or an SAS-SV screening cutoff, and the sample was drawn from a single institution and region. The findings therefore concern general college students with varying levels of problematic smartphone use tendencies and should not be generalized to clinically diagnosed populations or broader student groups. Future studies should recruit more diverse, multicenter samples and, where appropriate, use pre-specified screening thresholds or structured diagnostic assessments.

Fifth, smartphone-use tendencies and emotion regulation were assessed using self-report scales, which may be affected by social desirability, recall bias, self-evaluation errors, and measurement reactivity. Future studies should combine validated questionnaires with objective indicators such as app-use duration, screen-unlock frequency, smartphone-use records, behavioral tasks, and ecological momentary assessments.

Sixth, although heart-rate responses, Borg CR-10 ratings, movement quality, exercise completion, and discomfort symptoms were monitored during intervention delivery, the corresponding session-level records were not retained in a sufficiently complete and standardized format for aggregate quantitative analysis. Therefore, the protocol-specified intensity and dose targets should not be interpreted as quantitatively verified achieved values, and equivalence of the actual exercise intensity and dose across groups could not be established. Attendance and adverse-event records were the only complete quantitative indicators of adherence and intervention fidelity. Future studies should use standardized electronic fidelity-recording systems and report achieved exercise duration, intensity, completion, and protocol deviations at both the session and group levels.

## 5 Conclusion

Within this four-arm active-comparator randomized trial, the exercise modalities were associated with different magnitudes of pre–post change in SAS-SV scores and emotion regulation, with the combined condition showing the largest changes. Because participants were drawn from a general college-student population and were not recruited using diagnostic criteria or an SAS-SV screening cutoff, the findings concern problematic smartphone use tendencies and should not be interpreted as evidence regarding the treatment of clinically diagnosed smartphone addiction. In addition, because no non-exercise, wait-list, or attention-control condition was included, the study cannot determine whether exercise per se caused the observed changes. The exploratory relative indirect effects describe statistical associations among concurrent changes in cognitive reappraisal, expressive suppression, and SAS-SV scores and do not establish temporal or causal mediation. Future trials should incorporate appropriate control conditions, pre-specified screening criteria, repeated mediator assessments, and later outcome assessments.

## Data Availability

The raw data supporting the conclusions of this article will be made available by the authors, without undue reservation.
